# Canine endometrial and vaginal microbiomes reveal distinct and complex ecosystems

**DOI:** 10.1371/journal.pone.0210157

**Published:** 2019-01-07

**Authors:** C. C. Lyman, G. R. Holyoak, K. Meinkoth, X. Wieneke, K. A. Chillemi, U. DeSilva

**Affiliations:** 1 Department of Veterinary Clinical Sciences, Center for Veterinary Health Sciences, Oklahoma State University, Stillwater, Oklahoma, United States of America; 2 Department of Animal Science, Oklahoma State University, Stillwater, Oklahoma, United States Of America; University of Sydney Faculty of Veterinary Science, AUSTRALIA

## Abstract

The objective of this study was to characterize the normal microbiome of healthy canine vagina and endometrium and to determine the effect of the stage of estrous, on the resident microbiome. Cranial vaginal swabs and uterine biopsy samples were collected from twenty-five bitches in five different stages of estrous at elective ovariohysterectomy (OVH). Over 4 million reads of the V4 region of 16S rDNA gene were obtained and used for further analyses. A total of 317 genera belonging to 24 known phyla were identified. The endometrium was higher in bacterial diversity while the vagina was higher in richness. Proteobacteria, Bacteroidetes and Firmicutes were the most abundant phyla observed across all samples. *Hydrotalea*, *Ralstonia*, *and Fusobacterium* accounted for nearly 60% of the OTUs identified in the vagina while organisms identified in the endometrium were more evenly distributed. *Pseudomonas*, *Staphylococcus*, and *Corynebacterium* were the prominent genera in the endometrium. The microbiome of the endometrium was distinctly different from that of the vagina. There was large animal-to-animal variation. Other than the vaginal microbiome of bitches in estrus (i.e. in heat), there were no distinct clustering of the organisms based on the stage of estrous. These findings establish the presence of a resident microbiome of the endometrium throughout all stages of estrous cycle.

## Introduction

While it is established that the cranial vagina of mammals harbor a thriving microbial ecosystem, the uterus was long considered a sterile environment in order to sustain a successful pregnancy. This paradigm was recently challenged by the observation that the human placenta harbors a diverse microbiome [1, 2]. There is little information available on canine reproductive tract microbiota and all available data has been obtained using culture-based techniques [3–7]. It has been established that standard culture-based techniques fail to detect >90% of resident microflora [8], underestimate diversity [9], and overestimate the role of culturable bacteria [10].

A recent study utilizing culture-independent 16S ribosomal RNA (rRNA) sequence analysis of the bovine and ovine vaginal microbiota from ectocervicovaginal lavages revealed that cow and ewe vaginal microbiota are unique from previously described vaginal microbial ecosystems [11]. Similarly other studies specifically investigating the bovine uterine bacterial microbiota in healthy and metritic postpartum dairy cows found a significant diversity in the type and succession of bacterial communities related to clinical signs and in time progression [12–15].

Although the presence of commensal microorganisms in the uterine lumen is generally accepted in human medicine [2, 16, 17], the general consensus in canine medicine still is that the normal uterus is a sterile environment [18]. Many microbes are implicated in infertility and pregnancy loss [19, 20]. Culture of uterine swabs are performed routinely in bitches that fail to get pregnant with normal breed/cycle management and presence of any growth calls for antibiotic intervention [21]. As far as we are aware, this is the first comprehensive study of uterine and vaginal microbiota of normal, healthy bitches using a culture-independent technique. We provide essential and compelling evidence, that the canine uterus has a thriving microbial ecosystem and that treating a breeding bitch with antibiotics in the absence of abnormal diagnostic findings is counterproductive.

Here we describe a culture-independent approach to evaluate bacterial population structure and diversity of the cranial vagina and the endometrium of 25 clinically healthy bitches at various stages of estrous. We present a comparative analysis of the microbiomes of the vagina and the endometrium, organisms identified in both environments at both phylum and genus levels, and differences observed at different stages of estrous.

## Material and methods

### Animals

Fifty young, healthy bitches presented to Oklahoma State University, Boren Veterinary Medical Hospital for elective ovariohysterectomy (OVH) were utilized in sample collection. Dogs with clinical signs of estrus (i.e. in heat) were noted. None of the animals had recent antibiotic exposure. Bitches were induced and anesthetized using an accepted shelter protocol: Telazol-Torbugesic-Dexdomitor (Pfizer Animal Health, Parsippany, NJ) [22]. Once a level surgical plane of anesthesia was obtained, each patient had blood samples collected for hormonal assays. Samples for vaginal culture and cytology were collected using a standard technique [21]. Briefly, after placing a sterile otoscope cone beyond the vaginal vestibule, a sterile culturette swab (Becton Dickinson, Franklin Lakes, NJ) was passed through the speculum to contact the anterior vagina. The swab was placed inside of a sterile Eppendorf tube or sterile cryovial tube for transport to the lab. Vaginal epithelial cell samples were obtained by passing a vaginal cytologic brush through a vaginal speculum. A vaginal smear was prepared immediately and processed in Diff-Quick stain (Cole-Palmer, Vernon Hills, IL). The slides were evaluated to assist in classification of stages of estrous. Patients were then moved to the surgical suite for ovariohysterectomy. The reproductive tract was removed using sterile surgical technique. The uterine body was clamped and ligated proximal to the cervix. Two sections of uterine endometrial samples (1cm X1cm) were obtained using sterile Metzenbaum scissors and stored in individual sterile Eppendorf tubes or sterile cryovial tubes appropriate for liquid nitrogen cryopreservation of the endometrium until DNA extraction. Blood samples were evaluated using ELISA progesterone assay. Animal use and procedures were approved by the Oklahoma State University Institutional Animal Care and Use Committee (IACUC VM-14-42). All animals used in this study belonged to the Humane Society of Stillwater and were undergoing mandatory ovariohysterectomy prior to being offered for adoption. The director of the Humane Society approved of the collection of samples for the research described in this manuscript.

### Estrous cycle based group designation

Of the fifty animals that were utilized in sample collection, five animals whose samples clearly demarcated the stage of cycle were assigned to one of five groups based on the stage of the estrous cycle: pre-pubertal, anestrus, pro-estrus, estrus or diestrus (n = 5 per group) giving a total of twenty-five animals. Staging of the cycle was accomplished retrospectively via serum progesterone analysis and by interpretation of vaginal cytology (superficial cornification, or lack thereof) obtained immediately prior to the time of surgery, after which the samples were allocated to their respective groups. Pre-pubertal group was determined by age.

### DNA Isolation

Paired vaginal swab and endometrial biopsy samples from each animal were obtained for DNA extraction. 0.4g of tissue was ground up in liquid N_2_ and total DNA was extracted from individual samples using QiAamp DNA mini kit (Qiagen, Germantown, MD) and following manufacturer’s instructions. A 0.8% (wt/vol) low melting point agarose gel was used to measure DNA quality. DNA was purified by using the Agarose Gel DNA Purification Kit (TaKaRa Bio USA Inc., Mountainview, CA, USA). Purified DNA was quantified using a NanoDrop ND-1000 spectrophotometer (NanoDrop Technologies, Wilmington, DE, USA). We obtained 161–605 ng/μl DNA from endometrial biopsies and 58–128 ng/μl DNA from vaginal swabs. Each batch of DNA extractions and each new QiAamp DNA mini kit used were accompanied by a negative control consisting of H_2_O roughly at the same volume as the samples analyzed. Negative controls yielded 2–10 ng/μl DNA which was around the published minimal detection level of the NanoDrop instrument that was used for quantifying DNA. All DNA extractions were subjected to PCR amplification using 515F and 806R primer pair described below. All biopsy and swabs samples showed robust amplification of microbial DNA while the negative controls did not have a visible band after 35 cycles of amplification. Samples were stored at -20°C. Frozen DNA samples were sent to Molecular Research LLC for sequence analysis. Negative controls were not sequence analyzed as their concentration and quality was far below the threshold acceptable to the sequence provider.

### PCR amplification and DNA sequencing

A ~250 bp fragment from ribosomal v4 region was PCR-amplified from resulting DNA. PCR amplification and amplicon sequencing was performed by Molecular Research LLC (Mr. DNA), Shallowater, TX using their established protocols [23]. Briefly, an aliquot of 50 ng DNA was used as template for PCR amplification. The V4 region of 16S rRNA gene was amplified using the primer pair 515F (5’- GTGCCAGCMGCCGCGGTAA-3’) and 806R (5’-GGACTACHVGGGTWTCTAAT-3’) with added barcodes on the forward primer. DNA was amplified in a 28-cycle PCR using HotStarTaqPlus Master Mix Kit (Qiagen, USA) under the following conditions: 94°C for 3 minutes, followed by 28 cycles of 94°C for 30 seconds, 53°C for 40 seconds, and 72°C for 1 minute. Afterward, a final elongation step of 72°C for 5 minutes was performed.

A 2% agarose gel was used to determine the success of PCR amplification. DNA was purified using Qiagen Gel Extraction Kit (Qiagen, USA). Multiple barcoded samples were pooled in equal proportions based on their molecular weight and DNA concentrations. Pooled samples were purified with calibrated Ampure XP beads. The resulting pooled and purified PCR product was used to prepare Illumina sequencing library. The library was constructed using TruSeq DNA PCR-free sample preparation kit (Illumina Inc., San Diego, CA) following instructions and index codes were added. The library was quality tested on a Qubit 3.0 fluorometer (ThermoScientific, Waltham, MA) and an Agilent Bioanalyzer 2100 system (Agilent Technologies, Santa Clara, CA). The library was sequenced on Illumina HiSeq2500 platform (Illumina) and 250bp paired-end raw reads were generated.

### Bioinformatic analysis

Mothur 1.37 was used for data mining following the MiSeq standard operating procedure (SOP) [24]. Briefly, paired-end reads were assembled and assigned to each sample based on their unique barcode and then truncated by removing barcodes and primer sequences. Sequence reads that were over 273 bp in length, those that contained homoploymeric tracts of longer than 8 bp in length, those that had more than one mismatch against primer sequences, contained undetermined bases, ambiguities, or did not align to V4 hypervariable region were removed from the analysis. Qualified contigs were further processed with commands ‘trim.seqs’ and ‘pre.cluster’. Uchime was used to remove chimeric sequences [25]. Alignment of V4 region against SILVA rRNA ref nr123 [26] was performed using mothur (Needleman-Wunsch algorithm). Qualified sequences were classified into operational taxonomic units (OTUs) based on at least 97% similarity (OTU_0.03_). All sequences of OTU_0.03_ were assigned into taxonomic groups at the bootstrap threshold of 80%. Samples were randomly normalized to the sample with the least amount of sequencing reads to avoid sequencing bias. Alpha diversity was calculated using command ‘summary.single’ in mothur. Beta diversity was measured using Unifrac-based metrics generated with command ‘unifrac.weighted’ [27]. Raw sequencing data was submitted to Sequence Read Archive (SRA) at NCBI (access number SRP 115220).

### Statistical analyses

R 3.3.2 statistical analysis software [28] was used to perform one-way analyses of variance (one-way ANOVA), t-test, and Tukey’s honest significant difference (HSD) test. Differences were labeled as significant when the P value was <0.05. The unweighted pair group method with arithmetic mean(UPGMA) algorithm was performed using phangorn package in R [29]. The UPGMA tree was used to reveal the similarity of bacterial community composition.

Linear discriminant analysis (LDA) Effect Size (LEfSe) was used to determine the change in relative abundance of the bacterial community [30]. The non-parametric factorial Kruksal-Wallis (KW) sum-rank test was first used to determine taxa with significant abundant differences (*P*<0.05). The unpaired Wilcoxon rank-sum test was used to compare the significant abundant differences among taxa (*P*<0.05). Linear Discriminant Analysis was applied to calculate effective size of abundant differences. Neighbor-joining trees were generated using MEGAN Community Edition [31]. The Shannon diversity index was calculated using mothur and principal coordinate analyses (PCoA) was calculated based on the distance matrix.

## Results

**Fig 1:**
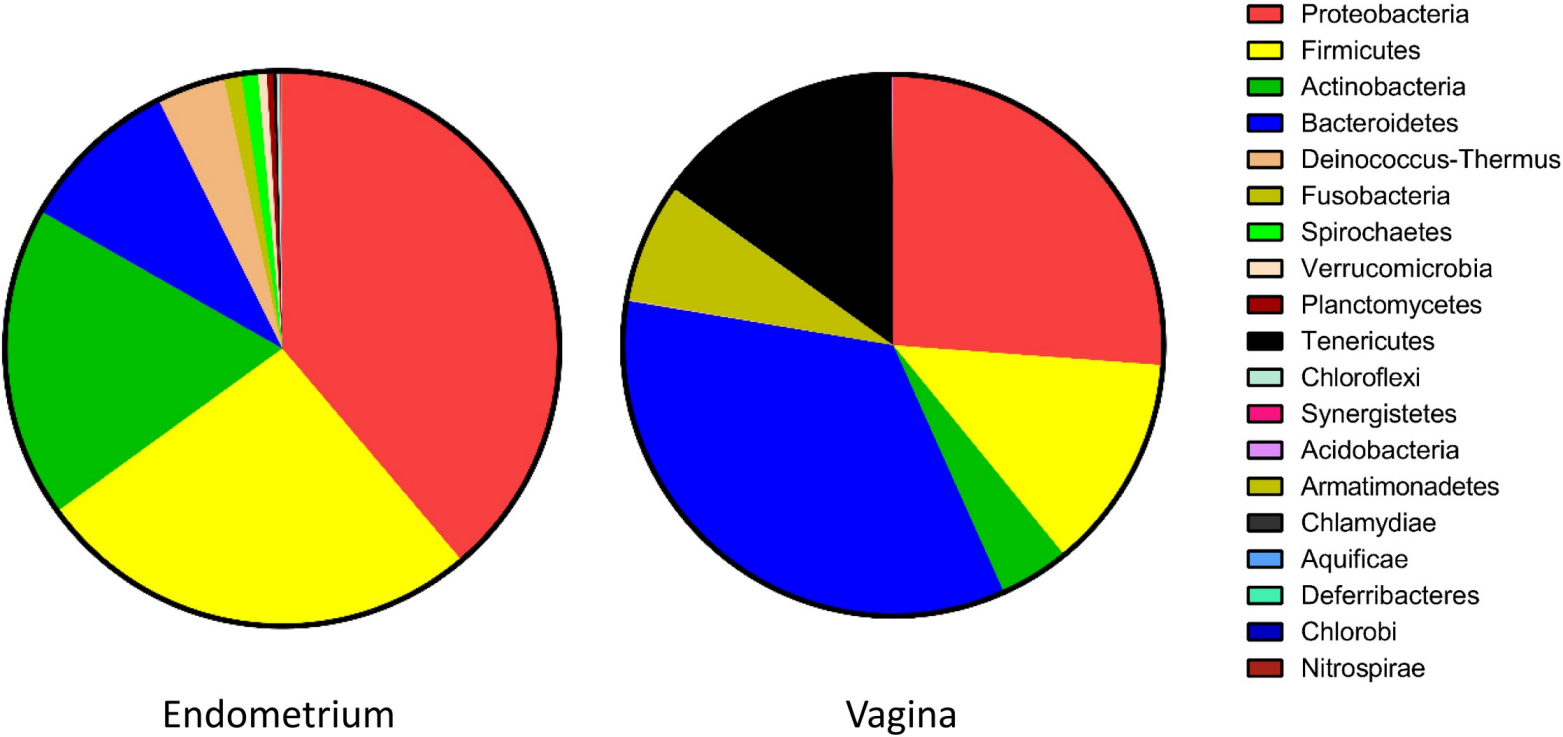
Bacterial community composition in canine endometrium and vagina. Community composition at phylum level.

16S rDNA sequencing resulted in 4,134,024 paired-end reads. After removal of ambiguous and low-quality reads, 3,527,169 reads remained resulting in 70,543±51,247 reads per sample. A taxonomy rarefaction curve (data not shown) indicated that the utilized sequencing depth was sufficient to saturate the bacterial diversity in canine endometrium and vagina. Next-generation sequencing with barcoded and pooled amplicons generates large variations in reads among samples. To eliminate sample size bias in downstream comparative analyses, we subsampled every animal/tissue to normalize the reads to that with the lowest number of reads [24, 32, 33]. After this process, 572,100 reads were selected for OTU classification. Of these, 562,971 reads were assigned to 24 known phyla and 384,974 sequence reads were classified into 317 known genera. Phylum Proteobacteria had the highest diversity with 120 OTUs, followed by Firmicutes (74 OTUs), Actinobacteria (46 OTUs), Bacteroidetes (38 OTUs), and Fusobacteria (5 OTUs). Proteobacteria was also the most abundant phylum at 24.23% followed by Bacteroidetes (17.32%), Firmicutes (14.4%), Actinobacteria (8.06%) Tenericutes (6.22%) and *Fusobacteria* (3.27%). Proteobacteria, Bacteroidetes, and Firmicutes, the three dominant phyla, accounted for 55.99% of the total bacterial community. Only three phyla, Proteobacteria, Bacteroidetes and Fusobacteria, were found in all 50 samples while Actinobacteria, Firmicutes and Tenericutes were observed in at least 47 samples. Bacteroidetes (34.3%), Proteobacteria (26.2%), Tenericutes (15%), and Firmicutes (12.9%) were the most prevalent phyla in the vagina while Proteobacteria (38.8%), Firmicutes (26.2%), Actinobacteria (18.2%), and Bacteroidetes (9.4%) were the most prevalent phyla in the uterus (Fig 1, Table 1). Of the 24 phyla identified in this study, Cyanobacteria, Elusimicrobia, Gemmatimonadetes, Lentisphaerae, and Thermotogae were only found in the vagina while Armatimonadetes, Chlamydiae, and Deferribacteres, were only identified in the uterus. Chloroflexi, Deinococcus-Thermus, Planctomycetes, Spirochaetes, Synergistetes, and Verrucomicrobia were extremely rare in the vagina (<5%). Firmicutes:Bacteroidetes ratio was lower in vagina compared to the uterus (0.38 and 1.61 respectively).

**Table 1:** Bacterial phyla identified in the endometrium and vagina. The numbers represent OTUs that could be classified into a known genus after subsampling (384,974 reads). Phyla ordered alphabetically. E = endometrium, V = vagina.

Phylum (n = 24)	E (n = 19)	V (n = 20)
Acidobacteria	54	102
Actinobacteria	32269	8685
Aquificae <phylum>	16	2
Armatimonadetes	50	0
Bacteroidetes	16706	71279
Chlamydiae	45	0
Chlorobi	1	5
Chloroflexi	319	14
Cyanobacteria	0	2
Deferribacteres <phylum>	5	0
Deinococcus-Thermus	7037	145
Elusimicrobia	0	1
Firmicutes	46560	26831
Fusobacteria	1806	14803
Gemmatimonadetes	0	4
Lentisphaerae	0	3
Nitrospirae	1	0
Planctomycetes	647	5
Proteobacteria	68861	54304
Spirochaetes	1725	32
Synergistetes	96	1
Tenericutes	357	31255
Thermotogae <phylum>	0	1
Verrucomicrobia	909	37

**Fig 2:**
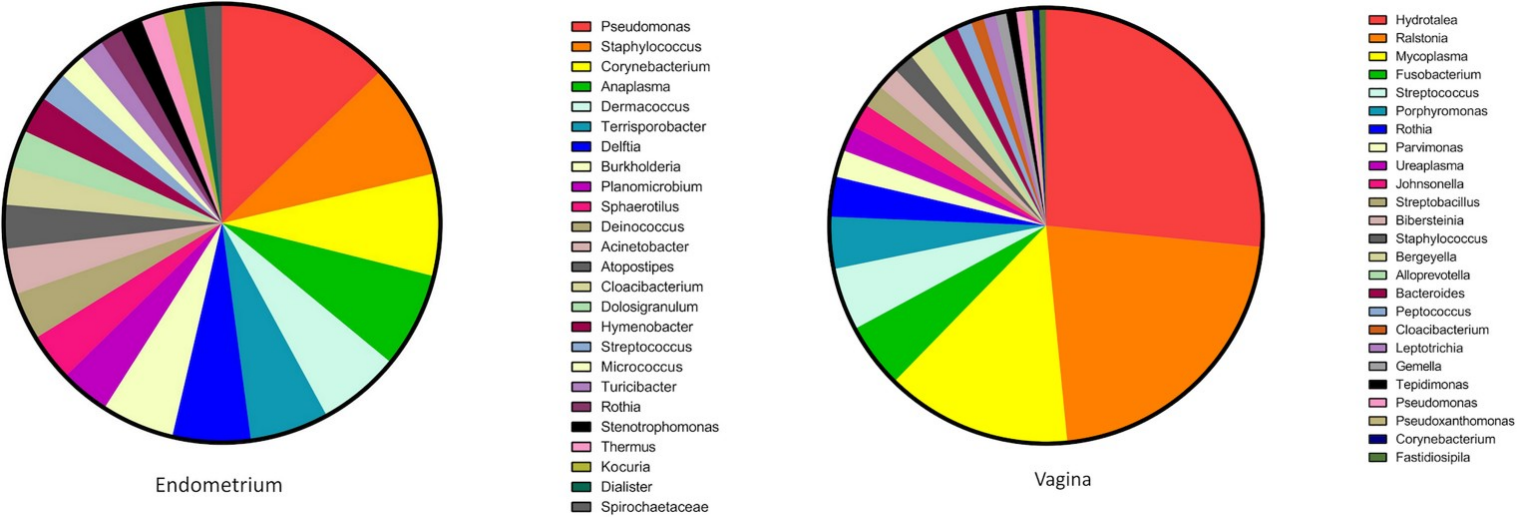
Bacterial community composition in canine endometrium and vagina. Community composition at genus level. Only the top 25 genera are depicted in the key for clarity.

At genus level (Fig 2 and S1 Table), 317 genera were identified in the endometrium and vagina (248 in the endometrium and 254 in the vagina); *Hydrotalea*, *Ralstonia*, and *Mycoplasma* accounted for 59.4% of the organisms identified in the vagina while the endometrium had a more evenly distributed microbiome. Microbiome of the endometrium was also more diverse than that of the vagina. *Hydrotalea*, *Ralstonia*, *Mycoplasma*, *Fusobacterium* and *Streptococcus* were the predominant species in the vagina; whereas *Pseudomonas*, *Staphylococcus*, and *Corynebacterium* were the predominant species in the uterus. Four genera, *Hydrotalea*, *Ralstonia*, *Pseudomonas*, and *Fusobacterium* existed ubiquitously across all samples accounting for 33.24% of the community while 92 (28.93%) genera were present only in single samples. Those represented 0.24% of the population. At genus level, we found the bacterial community in the vagina to be higher in richness and the uterus was more diverse (Table 2). The difference was not statistically significant in diversity, although richness between endometrial and vaginal microbiomes was significantly different (*P* = 0.0051).

**Table 2:** Diversity and Richness estimates of the endometrium and vagina at genus level.

	DIVERSITY	RICHNESS
**ENDOMETRIUM**	3.06 ± 0.65	7565.72 ± 2941.09
**VAGINA**	2.75 ± 0.512	10640.37 ± 2756.26

**Fig 3:**
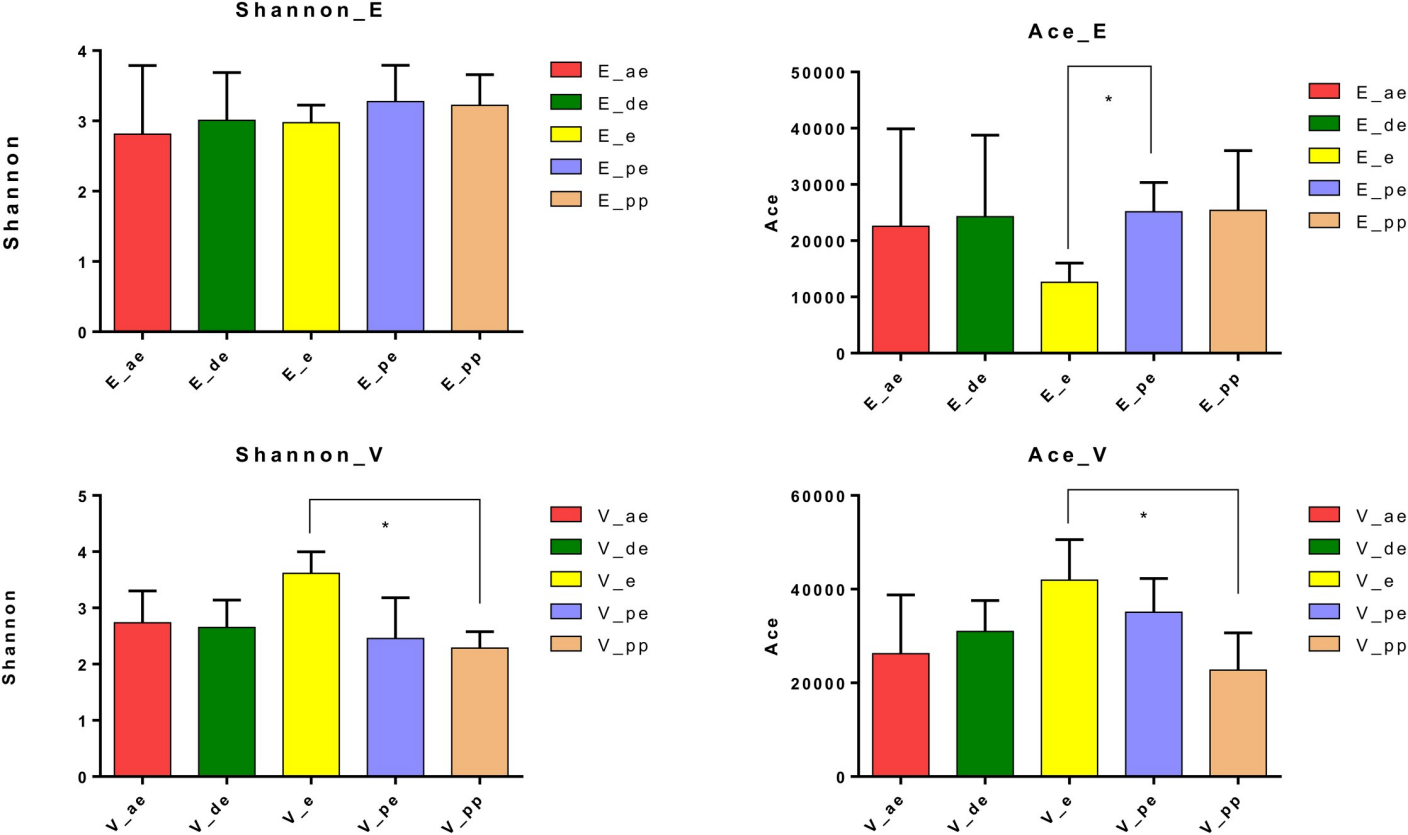
Variation in diversity (Shannon index) and richness (Ace) in endometrium (E) and vagina (V) at different stages of estrous cycle. Anestrus (ae) is depicted in red, diestrus (de) in green, estrus (e) in yellow, pro-estrus (pe) in purple, and pre-pubertal (pp) in dark beige.

We did not find significant differences in diversity (Shannon) and richness (Ace) among endometrial and vaginal samples between different stages of estrous in one-way ANOVA. However, Tukey’s HSD analysis shows a significant difference in diversity among vaginal microbiomes of animals in estrus (i.e. in heat) and those pre-pubertal (*P*<0.05). There was a significant difference in richness among endometrial samples at estrus and pro-estrus as well as vaginal samples in animals in estrus and those in pre-pubertal stage (Fig 3). Endometrium at estrus was the lowest in richness while the vagina at estrus and pro-estrus stages had the highest richness (Table 3).

**Table 3:** Diversity and richness estimations of endometrial and vaginal samples at different stages of estrous. (AE = anestrus, DE = diestrus, E = estrus, PE = pro-estrus and PP = pre-pubertal).

	DIVERISTY		RICHNESS	
	Endometrium	Vagina	Endometrium	Vagina
**AE**	2.81 ± 1.12	2.74 ± 0.61	7202.88 ± 5676.79	8598.18 ± 4667.98
**DE**	3.01 ± 0.76	2.65 ± 0.36	8210.13 ± 2819.41	9596.02 ± 878.71
**E**	2.97 ± 0.28	3.61 ± 0.42	4724.86 ± 1235.92	14495.97 ± 2126.36
**PE**	3.28 ± 0.59	2.46 ± 0.83	9618.37 ± 2299.23	12062.65 ± 3159.39
**PP**	3.22 ± 0.5	2.29 ± 0.34	8072.34 ± 2674.12	8449.01 ± 2948.87

**Fig 4:**
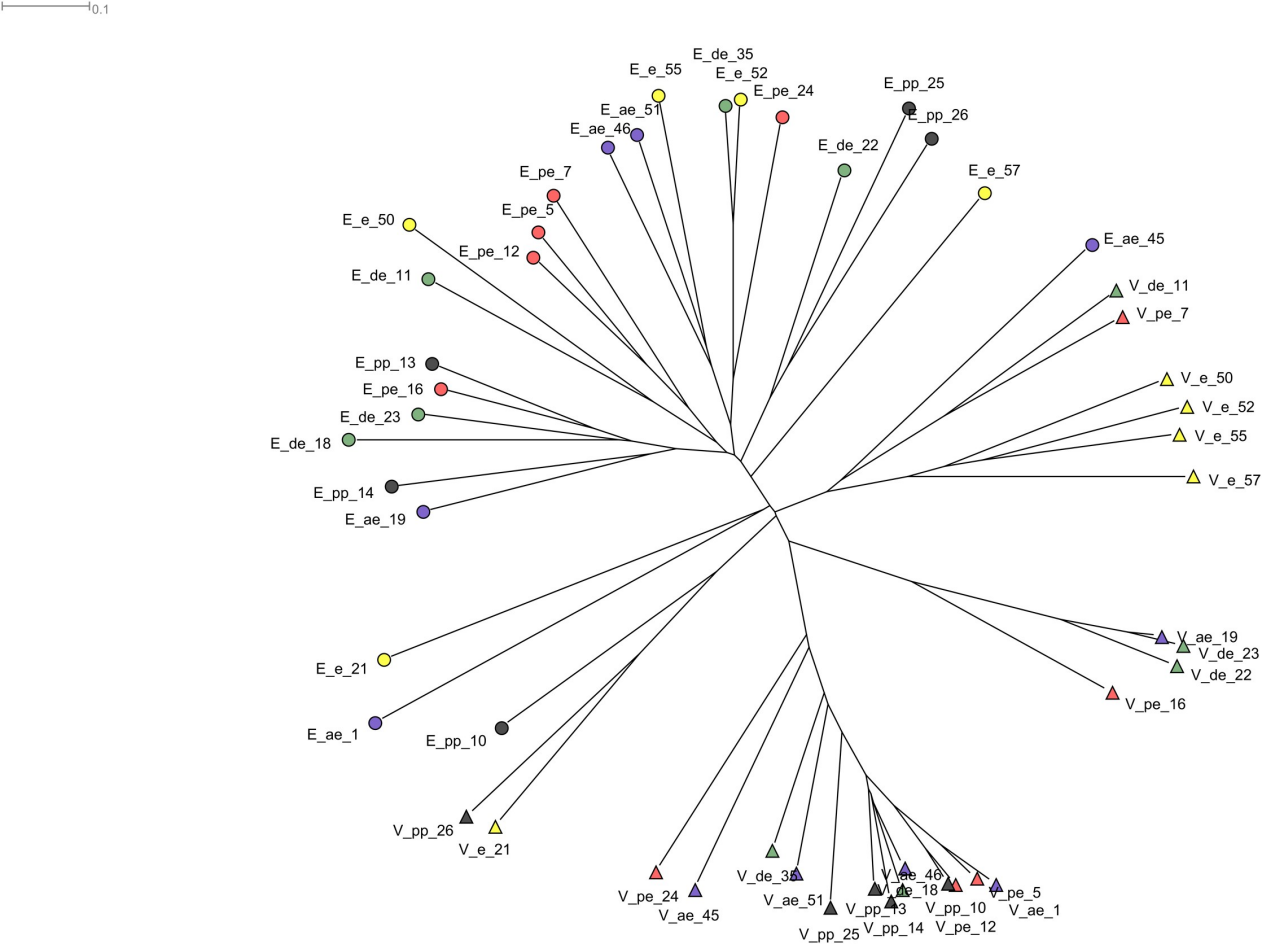
Neighbor-joining tree drawn at genus level. Circles represent endometrial samples while triangles are vaginal samples. Stages of estrous are color-coded. Anestrus (ae)-yellow, estrus (e)-green, diestrus (de)-green, pro-estrus (pe)-red, and pre-pubertal (pp)-gray.

**Fig 5:**
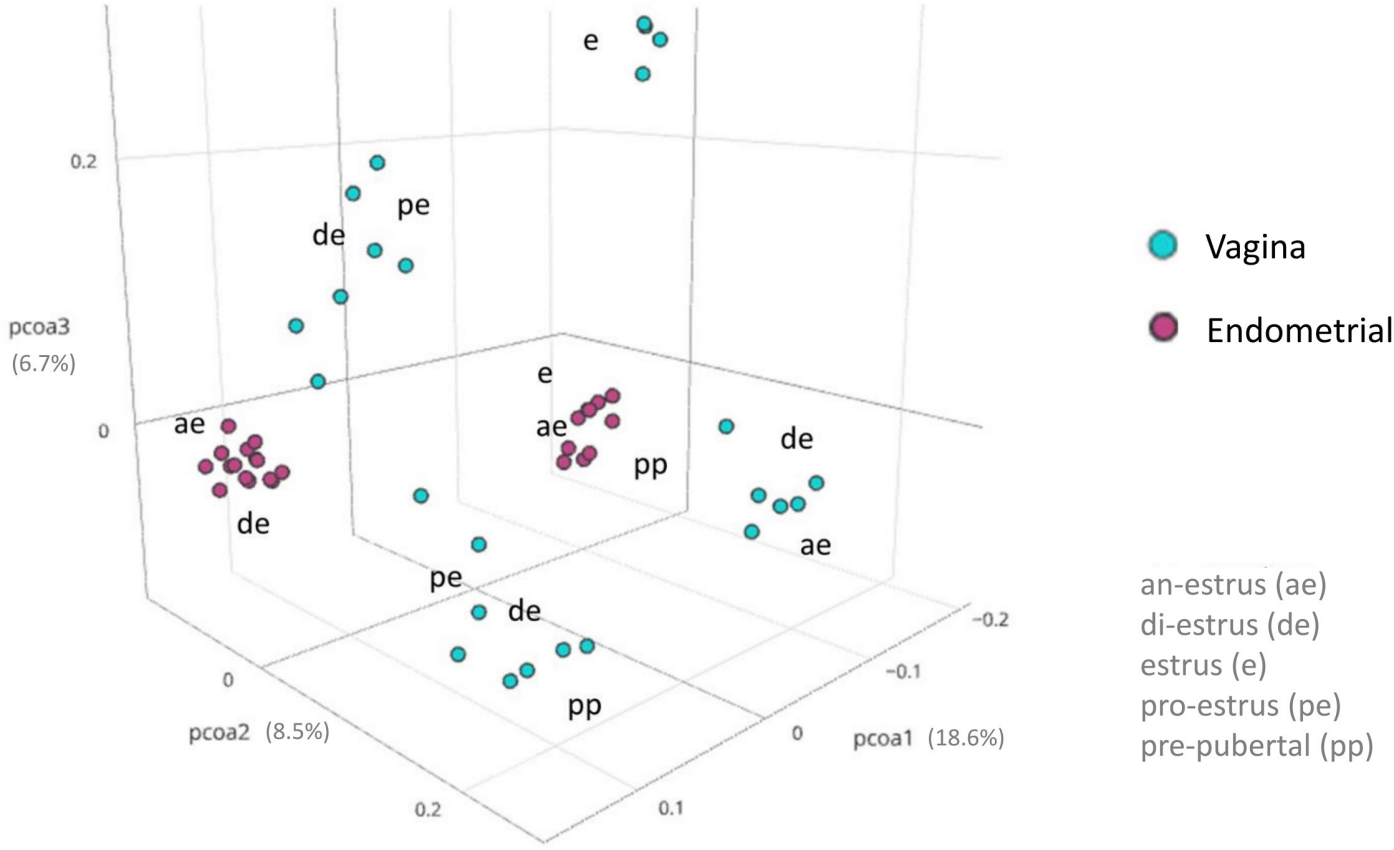
Principal Coordinates Analysis (PCoA). Distribution of bacterial communities based on the tissue and stage of estrous cycle. Community composition is measured using a UniFrac-weighted matrix. Vaginal samples are in blue and endometrial samples are in red. Anestrus-(ae), estrus-(e), diestrus-(de), pro-estrus-(pe) and pre-pubertal-(pp).

We evaluated the similarities between reproductive tract microbiota in bitches using the neighbor-joining tree approach of MEGAN [31] at genus level (Fig 4). As expected, there was a reasonably high inter-sample variability among samples. Vaginal samples clustered independent of the endometrial samples at all stages of estrous. The same was observed in Principal Coordinates Analysis (PCoA) (Fig 5). Other than vaginal samples from animals in estrus, there was little correlation between microbiomes and the stage of estrous. Vaginal samples at estrus were high in similarity and clustered strongly together. We did not observe similar clustering in any of the other stages of estrous in either tissue.

**Fig 6:**
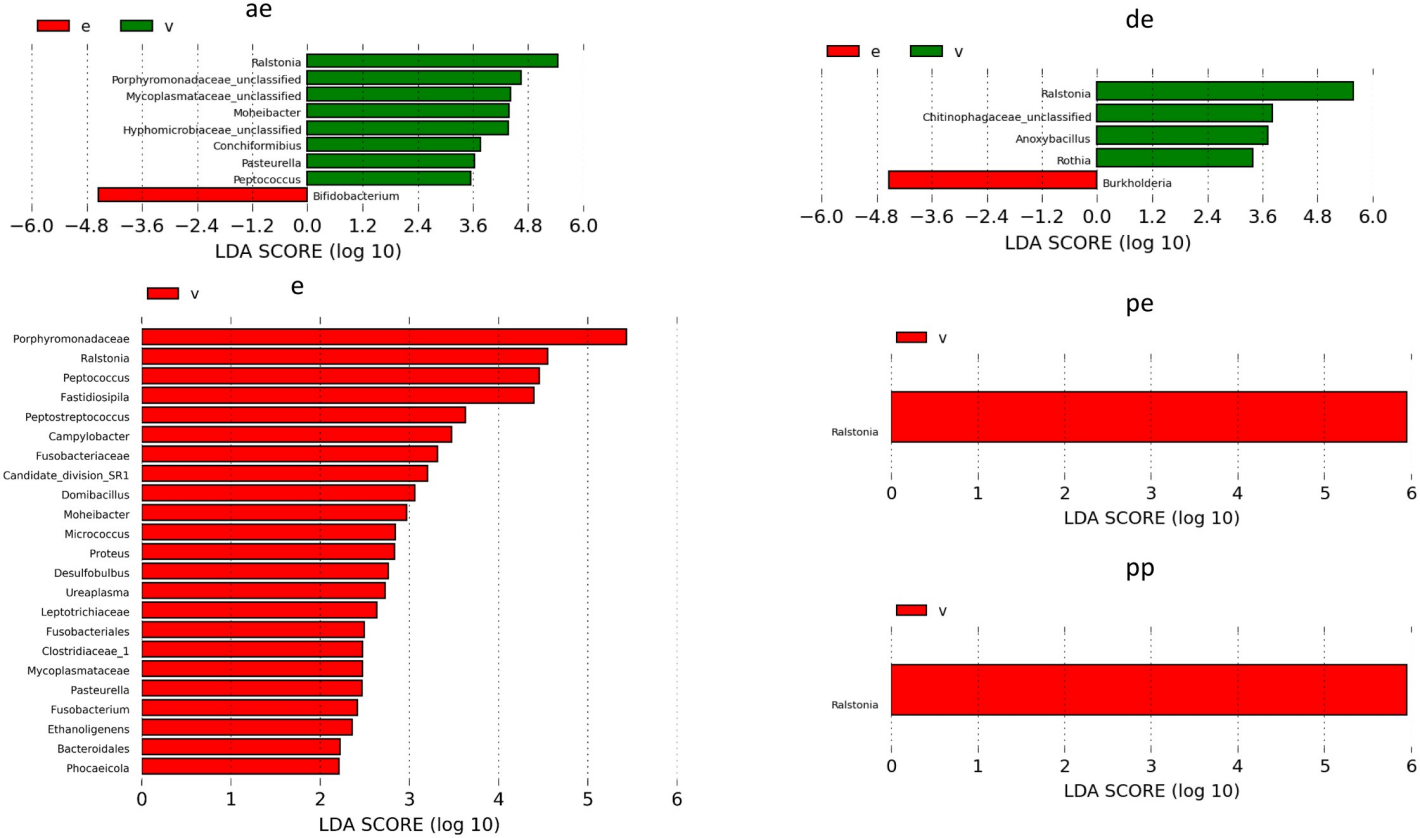
Linear discriminant analysis (LDA) effect size (LEfSe) analysis of endometrial (e) vs. vaginal (v) samples at different stages of estrous cycle. LefSe analysis identifies genera that differ significantly between the endometrium (e) and vagina (v). Relative abundance was considered significant when *P*<0.05 and LDA Score ≥2.0. Anestrus-(ae), estrus-(e), diestrus-(de), pro-estrus-(pe), and pre-pubertal-(pp).

We further confirmed the differentially abundant taxa by LEfSe, an algorithm for biomarker discovery that uses LDA to estimate the effect size of different taxa differentially represented in different environments (Fig 6). We used a cutoff LDA score of ≥2.0 for further analysis. *Ralstonia* was the most common differentially abundant species in the vagina scoring LDA scores >5.0 in all stages of estrous. Vagina at estrus had the largest number of genera (n = 24) that had LDA scores >2.0.

## Discussion

Although the mammalian vagina was known to harbor a rich microbial ecosystem, other than the well-described work done on the human reproductive tract [34–40], only a few non-human vaginal microbiomes have been described to date [11, 41–43]. The endometrium on the other hand, was considered a sterile environment [44, 45] until a few years ago when that paradigm was challenged by the observation that the human placenta possesses a small but diverse microbiome [1, 44]. Although the presence of a placental microbiome has been questioned recently due to the possibility of contamination [46, 47], the presence of a rich microbiome in the mammalian endometrium was established in several species [48–51]. A few studies have been performed exploring the microbiome of the canine reproductive tract [3, 6, 52–56], however these were all performed based on culture-based techniques that fail to identify a majority of organisms that inhabit a given ecosystem.

In this study, we sought to establish the vaginal and endometrial microbiomes of healthy bitches. Samples were collected from 50 bitches and the stage of estrous cycle was determined by serum progesterone analysis and vaginal cytology. Five animals were picked from each of the five stages of estrous for sequence analysis. All animals used in this study were stray or abandoned animals presented to a local animal shelter and, as such, we had no information on their exact age or breed. Most animals looked cross-bred. Although estimated age and the most probable breed composition of the animal was recorded, we did not select animals based on those two criteria. We were able to amplify 16S rDNA fragments from each sample. However, our methodology does not allow us to estimate the total amount of rDNA fragments amplified from each sample and as such, we cannot infer the density of microorganisms in any one of the samples. We found the vagina to be much higher in richness (number of different species) but lower in diversity (number of species and abundance of each species) compared to the endometrium (Table 2). The lower diversity is because 59.4% of the OTUs identified in the vagina belonged to three genera, *Ralstonia*, *Hydrotalea*, *and Mycoplasma* (S1 Table). The vagina at estrus (i.e. in heat) was the most diverse as well as the highest in richness (Table 3). The differences however were not statistically significant except between estrus and pre-pubertal stage (Fig 3). The lack of significant differences among vaginal microbiomes at main stages of estrous cycle is consistent with that seen in Göttingen minipigs [57]. Organisms identified as major occupants of the vaginal microbiome in this study are different from other published studies on canine vaginal microbiome. The most commonly isolated organisms in studies that are older than 10 years include; *Escherichia coli*, *Streptococcus canis*, *Pasteurella multocida*, *Staphylococcus aureus* and *Staphylococcus pseudintermedius* [3, 4, 58]. Of more recent studies, Maksimovic’ et al. [6] found *Staphylococcus spp*, *Streptococcus spp*, *Escherichia coli*, and *Proteus spp*. to be the most prevalent species and Hutchins et al. [7] found *Staphylococcus pseudintermedius*, *Streptococcus*, *Enterococcus*, and *Escherichia coli* to be the most prevalent species. Although we detected 9184 OTUs of *Streptococcus spp* they only represented 4.4% of the population (S1 Table). *Staphylococcus* represented 1.5% of the population, *Enterococcus represented* .006% of the population, and we only detected *Proteus* in the endometrium. Surprisingly, we did not detect any members of the genus *Escherichia* in our study. As noted before, all previous studies have been conducted using culture-based technologies making a direct comparison difficult. Culture-based technologies are biased towards organisms that grow well in culture media, aerobic organisms, and those that have a faster growth time. *Lactobacilli* is the most common organism identified in humans ranging from 70–100% of human vaginal microbiota [59–61]. We observed 76 OTUs representing *Lactobacillus spp* in the vagina and 25 OTUs in the endometrium. *Lactobacilli* in the canine vagina represented 0.03% of the population. The low abundance of *Lactobacilli* is consistent with what is observed in the cow and ewe reproductive tracts [11] and could be attributable to the near neutral to alkaline pH [62] of the canine vagina. *Hydrotalea* was the most prevalent genus in the vagina and accounted for over 25% of the organisms identified in the canine vagina. *Hydrotalea* belong to the family Bacteroidetes and is generally known as an aquatic species [63]. To our knowledge, other than low-level presence in a single study on antibiotic-induced changes in rat gut [64], *Hydrotalea* has never been reported in microbiomes of land-dwelling animals. *Ralstonia* accounted for 20.8% of organisms detected in the vagina. *Ralstonia* belongs to the phylum Proteobacteria and is not commonly associated with animals and no members of the genus is known to be pathogenic. It has never been reported in a healthy mammalian vagina although it has been reported in low concentrations in women that had levonorgestrel containing intrauterine devices placed in them (0.8% in vagina, 7.8% in uterus) [65] and in one study of women diagnosed with clinical vaginosis (0.8%) [66]. *Mycoplasma* (13.2%) was the third most abundant species. Although *Mycoplasma* was present in all 25 samples we tested, it is not considered a major organism in normal, healthy, vaginal flora of humans or animals [67]. However, it has been reported in bacterial vaginosis and other disease statuses in humans for decades [66, 68]. Watts et al., failed to culture *mycoplasma* from the reproductive tract of bitches at different stages of reproductive cycle [56], while it was isolated from bitches treated with ampicillin and trimethoprim-sulfamethoxazole [69]. We found high numbers of *Mycoplasma* ubiquitously present in all the samples we tested from the vagina and they were detected at much lower concentration (0.18%) in the endometrium suggesting that it is a commensal organism in the healthy canine reproductive tract.

In contrast to the vagina, we found the microbiome of the endometrium more evenly distributed. Although the phyla Proteobacteria, Firmicutes, Actinobacteria, and Bacteroidetes accounted for 92.6% of the 19 phyla identified in the endometrium (Table 1, Fig 1), the distribution of the organisms at genus level was more even (S1 Table, Fig 2). *Pseudomonas* (9.9%), *Staphylococcus* (6.5%) and *Campylobacter* (5.8%) were the most prevalent genera. Unlike in the vagina, no organism represented more than 10% of the population. This distribution is in stark contrast to endometrial microbiomes found in other species. In different human studies, endometrial microbiomes ranged from *Bacteroides* predominance [51], *Prevotella*, *Fusobacterium*, and *Jonquetella* Predominance [44, 70], to *Flavobacterium* and *Lactobacillus* predominance [49]. We found all these organisms with the exception of *Jonquetella* among our samples. However, they were present in very low abundance (S1 Table). In dairy cattle, most abundant genera were *Fusobacterium*, *Bacteroides*, *Coxiella*, *Porphyromonas*, and class Gammaproteobacteria [71, 72]. We did not observe any *Coxiella* in bitches. We observed similar differences in vaginal microbiomes as well suggesting that core endometrial and vaginal microbiomes tend to be species-specific. A few attempts have been made at isolating microbes from the canine endometrium using culture-based techniques [3–6]. The success rate at isolating bacteria ranged from 3.8% [4] to 62.5% [6] proving that most endometrial microbes are not amenable to culture using standard techniques. Most prevalent organisms isolated in these studies were *Staphylococcus*, *Mycoplasma* and in one study *Streptococcus* [4]. *Staphylococcus* was the second-most abundant organism in our study whereas *Mycoplasma* and *Streptococcus* were much rarer. *Staphylococcus* and *Streptococcus* are also easily cultured under standard technology explaining their abundance in culture-based studies.

Intriguingly, the most abundant organisms in the canine endometrium belonged to the genus *Pseudomonas* and was identified in all endometrial samples. *Pseudomonas spp* is often associated with pyometra in bitches [73] but has not been reported in a healthy endometrium. The SILVA database [26] was used to classify OTUs and does not allow taxonomic classification at species level. Although indirect evidence suggests that the genus *Pseudomonas* in the canine endometrium consists of several species, the exact identity of the organisms remain unknown. *Staphylococcus*, the genus with second highest abundance in the endometrium has been isolated from the canine endometrium in previous studies [3, 5, 6]. *Campylobacter* represented 5.8% of organisms identified in the endometrium and seems to be a major representative of the canine endometrial microbiome. Although *Campylobacter* associated reproductive tract pathology has not been reported in bitches, different species of *Campylobacter* has been implicated in abortions in cattle and sheep [74, 75].

The prevalent explanation for the presence of microbes in the uterus was that they always are introduced as an ascending infection from the vagina through an open cervix during pro-estrus and estrus. To test this hypothesis, we compared the vaginal and uterine microbiomes of the five bitches in estrus (Fig 6). Contrary to the prevailing notion, we did not find a correlation between the vaginal and uterine microbiomes of any of the bitches suggesting that the endometrium has its own resident microbiome and it was not established as an ascending infection from the vagina [76, 77].

Since the focus of this study was to ascertain the presence of an endometrial microbiome of healthy bitches and to compare the microbiome across different stages of estrous, we did not analyze the effect of breed and age of the animal (other than pre-pubertal animals). The source of our experimental animals (local humane society) would have made reliable age and breed estimates difficult. Although it is possible for breed differences in reproductive tract microbiomes to exist, many studies have found no differences in resident microbiomes of animals of different breeds housed in similar conditions [78–80]. We found the diversity and richness of microbiomes of pre-pubertal animals to be significantly different from older animals (Fig 3). Both breeds and ages were rough phenotypic estimates as all of these animals were obtained from a local Humane Society and most looked crossbred. We were intrigued to find a rich, diverse uterine microbiome even in the youngest pre-pubertal animals used in the study.

## Conclusions

From this study, we conclude that both the endometrium and the vagina have rich microbial ecosystems. The endometrium is much more diverse than the vagina. The endometrial microbiome does not change with the stage of the estrous cycle. Whereas the vaginal environment in estrus (i.e. in heat) is significantly different from other stages of estrous. There is no age or breed effect, while significant animal-to-animal variation in endometrial and vaginal microbiome does exist. We conclude from our results as well as past studies that culture-based systems miss the great diversity present in both diseased and healthy reproductive tracts. Given this diversity, there is much to be studied relative to the intra-microbiota interactions and species-intrinsic factors that may be more relevant to maintaining a state of balance and health and that an imbalance of these factors maybe more important in the development of uterine disease than the abundance of any given bacterial species.

## Supporting information

S1 Table Bacterial genera identified in canine endometrium and vagina. Column A is sorted by prevalence in the vagina and Column B is sorted by prevalence in the endometrium. 317 known genera were identified among the samples. 254 genera were present in the vagina and 248 were present in the endometrium.
(DOCX)
