# Supplementary material for: Canine endometrial and vaginal microbiomes reveal distinct and complex ecosystems
Source: PLoS One. 2019 Jan 7;14(1):e0210157. doi: 10.1371/journal.pone.0210157 (PMC6322750; doi:10.1371/journal.pone.0210157)
Supplement: S1 Table — Column A is sorted by prevalence in the vagina and Column B is sorted by prevalence in the endometrium. 317 known genera were identified among the samples. 254 genera were present in the vagina and 248 were present in the endometrium. (DOCX) [file pone.0210157.s001.docx]

**S1 Table.**

| **A** | | | **B** | | |
| --- | --- | --- | --- | --- | --- |
| **Genus** | **Vagina** | **Endometrium** | **Genus** | **Vagina** | **Endometrium** |
| Hydrotalea | 52567 | 410 | Pseudomonas | 1220 | 17540 |
| Ralstonia | 43268 | 1714 | Staphylococcus | 3038 | 11547 |
| Mycoplasma | 27484 | 324 | Corynebacterium | 1045 | 10266 |
| Fusobacterium | 9584 | 1646 | Anaplasma | 106 | 9611 |
| Streptococcus | 9184 | 3027 | Dermacoccus | 264 | 8301 |
| Porphyromonas | 7618 | 747 | Terrisporobacter | 54 | 7955 |
| Rothia | 5883 | 2340 | Delftia | 374 | 7892 |
| Parvimonas | 3942 | 185 | Burkholderia | 170 | 7338 |
| Ureaplasma | 3769 | 31 | Planomicrobium | 46 | 4982 |
| Johnsonella | 3608 | 126 | Sphaerotilus | 86 | 4802 |
| Streptobacillus | 3353 | 31 | Deinococcus | 119 | 4796 |
| Bibersteinia | 3345 | 31 | Acinetobacter | 531 | 4587 |
| Staphylococcus | 3038 | 11547 | Atopostipes | 481 | 4408 |
| Bergeyella | 2993 | 78 | Cloacibacterium | 1893 | 3837 |
| Alloprevotella | 2562 | 107 | Dolosigranulum | 50 | 3736 |
| Bacteroides | 2322 | 97 | Hymenobacter | 27 | 3732 |
| Peptococcus | 2180 | 69 | Streptococcus | 9184 | 3027 |
| Cloacibacterium | 1893 | 3837 | Micrococcus | 104 | 2642 |
| Leptotrichia | 1841 | 129 | Turicibacter | 246 | 2420 |
| Gemella | 1547 | 109 | Rothia | 5883 | 2340 |
| Tepidimonas | 1515 | 311 | Stenotrophomonas | 310 | 2253 |
| Pseudomonas | 1220 | 17540 | Thermus | 26 | 2241 |
| Pseudoxanthomonas | 1159 | 66 | Kocuria | 207 | 2168 |
| Corynebacterium | 1045 | 10266 | Dialister | 44 | 2083 |
| Fastidiosipila | 956 | 8 | Spirochaetaceae | 32 | 1725 |
| Moheibacter | 896 | 1423 | Ralstonia | 43268 | 1714 |
| Actinomyces | 699 | 645 | Fusobacterium | 9584 | 1646 |
| Acinetobacter | 531 | 4587 | Brevibacterium | 25 | 1457 |
| Atopostipes | 481 | 4408 | Ehrlichia | 2 | 1437 |
| Moraxella | 465 | 999 | Moheibacter | 896 | 1423 |
| Delftia | 374 | 7892 | Gordonia | 120 | 1117 |
| Peptostreptococcus | 354 | 85 | Cesiribacter | 11 | 1071 |
| Necropsobacter | 311 | 14 | Moraxella | 465 | 999 |
| Stenotrophomonas | 310 | 2253 | Anoxybacillus | 11 | 964 |
| Dermacoccus | 264 | 8301 | Campylobacter | 193 | 953 |
| Turicibacter | 246 | 2420 | Arsenicicoccus | 31 | 917 |
| Sphingobacterium | 235 | 699 | Veillonella | 21 | 914 |
| Chelatococcus | 216 | 0 | Opitutus | 25 | 853 |
| Kocuria | 207 | 2168 | Zoogloea | 17 | 813 |
| Macrococcus | 195 | 2 | Tannerella | 19 | 805 |
| Campylobacter | 193 | 953 | Azospira | 7 | 776 |
| Burkholderia | 170 | 7338 | Alcaligenes | 29 | 768 |
| Pasteurella | 164 | 2 | Thioclava | 10 | 756 |
| Conchiformibius | 160 | 189 | Porphyromonas | 7618 | 747 |
| Bifidobacterium | 158 | 178 | Sphingobacterium | 235 | 699 |
| Ezakiella | 133 | 138 | Actinomyces | 699 | 645 |
| Gordonia | 120 | 1117 | Draconibacterium | 2 | 634 |
| Deinococcus | 119 | 4796 | Peptoniphilus | 15 | 604 |
| Anaplasma | 106 | 9611 | Dysgonomonas | 2 | 567 |
| Micrococcus | 104 | 2642 | Hydrogenophilus | 21 | 509 |
| Nitrobacter | 98 | 1 | Prevotella | 4 | 502 |
| Abiotrophia | 87 | 7 | Methylophilus | 37 | 501 |
| Sphaerotilus | 86 | 4802 | Proteiniphilum | 14 | 484 |
| Lactobacillus | 76 | 25 | Alcanivorax | 4 | 440 |
| Leuconostoc | 75 | 0 | Domibacillus | 31 | 432 |
| Bryobacter | 62 | 3 | Barnesiella | 5 | 427 |
| Janibacter | 58 | 338 | Paenibacillus | 12 | 426 |
| Terrisporobacter | 54 | 7955 | Hydrotalea | 52567 | 410 |
| Dolosigranulum | 50 | 3736 | Janthinobacterium | 25 | 348 |
| Planomicrobium | 46 | 4982 | Janibacter | 58 | 338 |
| Dialister | 44 | 2083 | Desulfobacula | 1 | 336 |
| Arcobacter | 44 | 1 | Mycoplasma | 27484 | 324 |
| Chryseobacterium | 42 | 267 | Desemzia | 0 | 323 |
| Lachnoclostridium | 42 | 3 | Thermomicrobia | 13 | 318 |
| Sutterella | 42 | 1 | Tepidimonas | 1515 | 311 |
| Blastocatella | 39 | 6 | Singulisphaera | 1 | 297 |
| Megasphaera | 39 | 0 | Phocaeicola | 3 | 292 |
| Methylophilus | 37 | 501 | Flavisolibacter | 7 | 282 |
| Alteromonas | 36 | 0 | Chryseobacterium | 42 | 267 |
| Psychrobacter | 33 | 0 | Rickettsiaceae | 0 | 261 |
| Spirochaetaceae | 32 | 1725 | Ornithinibacillus | 1 | 259 |
| Ureibacillus | 32 | 1 | Actinomycetospora | 16 | 254 |
| Pedobacter | 32 | 0 | Intestinibacter | 7 | 253 |
| Arsenicicoccus | 31 | 917 | Nocardioides | 6 | 253 |
| Domibacillus | 31 | 432 | Anaerococcus | 1 | 241 |
| Proteus | 31 | 0 | Desulfobulbus | 5 | 240 |
| Alcaligenes | 29 | 768 | Psychromonas | 1 | 237 |
| Hymenobacter | 27 | 3732 | Tepidiphilus | 16 | 234 |
| Blautia | 27 | 1 | Devosia | 8 | 225 |
| Thermus | 26 | 2241 | Thermicanus | 17 | 209 |
| Brevibacterium | 25 | 1457 | Neisseria | 6 | 207 |
| Opitutus | 25 | 853 | Rhodococcus | 4 | 204 |
| Janthinobacterium | 25 | 348 | Cellvibrio | 10 | 192 |
| Granulicatella | 25 | 189 | Conchiformibius | 160 | 189 |
| Filifactor | 24 | 0 | Granulicatella | 25 | 189 |
| Rheinheimera | 24 | 0 | Parvimonas | 3942 | 185 |
| Geobacillus | 22 | 170 | Bordetella | 1 | 184 |
| Veillonella | 21 | 914 | Planctomyces | 1 | 184 |
| Hydrogenophilus | 21 | 509 | Steroidobacter | 1 | 180 |
| Pleomorphomonas | 21 | 0 | Bifidobacterium | 158 | 178 |
| Tannerella | 19 | 805 | Brevundimonas | 1 | 176 |
| Zoogloea | 17 | 813 | Geobacillus | 22 | 170 |
| Thermicanus | 17 | 209 | Nakamurella | 1 | 167 |
| Peptoclostridium | 17 | 19 | Desulfovibrio | 12 | 166 |
| Anaerostipes | 17 | 1 | Pirellula | 1 | 165 |
| Proteocatella | 17 | 1 | Neorickettsia | 1 | 163 |
| Sporosarcina | 17 | 0 | Slackia | 3 | 159 |
| Actinomycetospora | 16 | 254 | Ethanoligenens | 6 | 149 |
| Tepidiphilus | 16 | 234 | Microvirga | 1 | 147 |
| Peptoniphilus | 15 | 604 | Sanguibacter | 0 | 145 |
| Allobaculum | 15 | 20 | Ezakiella | 133 | 138 |
| Proteiniphilum | 14 | 484 | Undibacterium | 0 | 134 |
| Daeguia | 14 | 24 | Schlegelella | 4 | 130 |
| Faecalibacterium | 14 | 0 | Leptotrichia | 1841 | 129 |
| Sebaldella | 14 | 0 | Aquimonas | 1 | 128 |
| Thermomicrobia | 13 | 318 | Iamia | 2 | 127 |
| Olsenella | 13 | 102 | Myroides | 2 | 127 |
| Enterococcus | 13 | 5 | Johnsonella | 3608 | 126 |
| Acidibacter | 13 | 0 | Methylobacterium | 2 | 126 |
| Caulobacter | 13 | 0 | Aeromonas | 7 | 121 |
| Paenibacillus | 12 | 426 | Shimazuella | 0 | 120 |
| Desulfovibrio | 12 | 166 | Kineococcus | 1 | 117 |
| Catonella | 12 | 64 | Rubellimicrobium | 2 | 112 |
| Catenibacterium | 12 | 0 | Gemella | 1547 | 109 |
| Erysipelothrix | 12 | 0 | Alloprevotella | 2562 | 107 |
| Cesiribacter | 11 | 1071 | Olsenella | 13 | 102 |
| Anoxybacillus | 11 | 964 | Brachybacterium | 3 | 99 |
| Polynucleobacter | 11 | 15 | Fluviicola | 1 | 98 |
| Akkermansia | 11 | 9 | Bacteroides | 2322 | 97 |
| Sneathia | 11 | 0 | Pyramidobacter | 1 | 96 |
| Thioclava | 10 | 756 | Haliangium | 1 | 87 |
| Cellvibrio | 10 | 192 | Peptostreptococcus | 354 | 85 |
| Craurococcus | 10 | 52 | Selenomonas | 1 | 82 |
| Fusibacter | 10 | 0 | Bergeyella | 2993 | 78 |
| Oligoflexales | 9 | 42 | Gaiella | 1 | 76 |
| Quadrisphaera | 9 | 0 | Hyphomicrobium | 0 | 74 |
| Devosia | 8 | 225 | Peptococcus | 2180 | 69 |
| Helicobacter | 8 | 6 | Pseudoxanthomonas | 1159 | 66 |
| Megamonas | 8 | 0 | Catonella | 12 | 64 |
| Azospira | 7 | 776 | Mogibacterium | 1 | 59 |
| Flavisolibacter | 7 | 282 | Dietzia | 3 | 56 |
| Intestinibacter | 7 | 253 | Bosea | 0 | 55 |
| Aeromonas | 7 | 121 | Craurococcus | 10 | 52 |
| Lysobacter | 7 | 1 | Oribacterium | 1 | 52 |
| Adhaeribacter | 7 | 0 | Armatimonadetes | 0 | 50 |
| Cardiobacteriaceae | 7 | 0 | Mycobacterium | 3 | 48 |
| Morganella | 7 | 0 | Prosthecobacter | 0 | 47 |
| Nocardioides | 6 | 253 | Asaccharospora | 0 | 45 |
| Neisseria | 6 | 207 | Holophagaceae | 0 | 45 |
| Ethanoligenens | 6 | 149 | Simkaniaceae | 0 | 45 |
| Sphingobium | 6 | 20 | Oligoflexales | 9 | 42 |
| Alistipes | 6 | 3 | Ureaplasma | 3769 | 31 |
| Barnesiella | 5 | 427 | Streptobacillus | 3353 | 31 |
| Desulfobulbus | 5 | 240 | Bibersteinia | 3345 | 31 |
| Enhydrobacter | 5 | 30 | Acidimicrobiaceae | 0 | 31 |
| Chlorobiales | 5 | 1 | Macromonas | 0 | 31 |
| Christensenellaceae | 5 | 0 | Enhydrobacter | 5 | 30 |
| Collinsella | 5 | 0 | Lactobacillus | 76 | 25 |
| Defluviitaleaceae | 5 | 0 | Daeguia | 14 | 24 |
| Ornithinimicrobium | 5 | 0 | Allobaculum | 15 | 20 |
| Phascolarctobacterium | 5 | 0 | Sphingobium | 6 | 20 |
| Prevotella | 4 | 502 | Peptoclostridium | 17 | 19 |
| Alcanivorax | 4 | 440 | Sulfurihydrogenibium | 0 | 16 |
| Rhodococcus | 4 | 204 | Polynucleobacter | 11 | 15 |
| Schlegelella | 4 | 130 | Tersicoccus | 2 | 15 |
| Mannheimia | 4 | 1 | Necropsobacter | 311 | 14 |
| Cellulomonas | 4 | 0 | Planifilum | 0 | 13 |
| Duganella | 4 | 0 | Stakelama | 0 | 13 |
| Gemmatimonas | 4 | 0 | Solobacterium | 1 | 11 |
| Phocaeicola | 3 | 292 | Akkermansia | 11 | 9 |
| Slackia | 3 | 159 | Sphingomonas | 2 | 9 |
| Brachybacterium | 3 | 99 | Millisia | 0 | 9 |
| Dietzia | 3 | 56 | Fastidiosipila | 956 | 8 |
| Mycobacterium | 3 | 48 | Leminorella | 1 | 8 |
| Dyadobacter | 3 | 1 | Propionibacterium | 1 | 8 |
| Anaerobiospirillum | 3 | 0 | Abiotrophia | 87 | 7 |
| Bhargavaea | 3 | 0 | Acaricomes | 0 | 7 |
| Holdemanella | 3 | 0 | Blastocatella | 39 | 6 |
| Lentisphaerae | 3 | 0 | Helicobacter | 8 | 6 |
| Novosphingobium | 3 | 0 | Azorhizophilus | 1 | 6 |
| Promicromonospora | 3 | 0 | Filibacter | 1 | 6 |
| Thermoanaerobacterium | 3 | 0 | Enterococcus | 13 | 5 |
| Ehrlichia | 2 | 1437 | Paenirhodobacter | 2 | 5 |
| Draconibacterium | 2 | 634 | Cruoricaptor | 0 | 5 |
| Dysgonomonas | 2 | 567 | Fodinicola | 0 | 5 |
| Iamia | 2 | 127 | Mucispirillum | 0 | 5 |
| Myroides | 2 | 127 | Yimella | 1 | 4 |
| Methylobacterium | 2 | 126 | Devriesea | 0 | 4 |
| Rubellimicrobium | 2 | 112 | Fontibacillus | 0 | 4 |
| Tersicoccus | 2 | 15 | Bryobacter | 62 | 3 |
| Sphingomonas | 2 | 9 | Lachnoclostridium | 42 | 3 |
| Paenirhodobacter | 2 | 5 | Alistipes | 6 | 3 |
| Phycisphaeraceae | 2 | 1 | Tissierella | 1 | 3 |
| Sarcina | 2 | 1 | Macrococcus | 195 | 2 |
| Solirubrobacter | 2 | 1 | Pasteurella | 164 | 2 |
| Acholeplasma | 2 | 0 | Desulfatiglans | 1 | 2 |
| Desulfomicrobium | 2 | 0 | Oxalicibacterium | 1 | 2 |
| Pontibacter | 2 | 0 | Parabacteroides | 1 | 2 |
| Synechococcus | 2 | 0 | Anaeroplasma | 0 | 2 |
| Venenivibrio | 2 | 0 | Bdellovibrio | 0 | 2 |
| Desulfobacula | 1 | 336 | Fibrella | 0 | 2 |
| Singulisphaera | 1 | 297 | Nitrosococcus | 0 | 2 |
| Ornithinibacillus | 1 | 259 | Pseudonocardia | 0 | 2 |
| Anaerococcus | 1 | 241 | Nitrobacter | 98 | 1 |
| Psychromonas | 1 | 237 | Arcobacter | 44 | 1 |
| Bordetella | 1 | 184 | Sutterella | 42 | 1 |
| Planctomyces | 1 | 184 | Ureibacillus | 32 | 1 |
| Steroidobacter | 1 | 180 | Blautia | 27 | 1 |
| Brevundimonas | 1 | 176 | Anaerostipes | 17 | 1 |
| Nakamurella | 1 | 167 | Proteocatella | 17 | 1 |
| Pirellula | 1 | 165 | Lysobacter | 7 | 1 |
| Neorickettsia | 1 | 163 | Chlorobiales | 5 | 1 |
| Microvirga | 1 | 147 | Mannheimia | 4 | 1 |
| Aquimonas | 1 | 128 | Dyadobacter | 3 | 1 |
| Kineococcus | 1 | 117 | Phycisphaeraceae | 2 | 1 |
| Fluviicola | 1 | 98 | Sarcina | 2 | 1 |
| Pyramidobacter | 1 | 96 | Solirubrobacter | 2 | 1 |
| Haliangium | 1 | 87 | Gardnerella | 1 | 1 |
| Selenomonas | 1 | 82 | Klugiella | 1 | 1 |
| Gaiella | 1 | 76 | Odoribacter | 1 | 1 |
| Mogibacterium | 1 | 59 | Oscillibacter | 1 | 1 |
| Oribacterium | 1 | 52 | Roseburia | 1 | 1 |
| Solobacterium | 1 | 11 | Senegalimassilia | 1 | 1 |
| Leminorella | 1 | 8 | Thalassospira | 1 | 1 |
| Propionibacterium | 1 | 8 | Actinobacillus | 0 | 1 |
| Azorhizophilus | 1 | 6 | Allisonella | 0 | 1 |
| Filibacter | 1 | 6 | Arcticibacter | 0 | 1 |
| Yimella | 1 | 4 | Azomonas | 0 | 1 |
| Tissierella | 1 | 3 | Azospirillum | 0 | 1 |
| Desulfatiglans | 1 | 2 | Caldimonas | 0 | 1 |
| Oxalicibacterium | 1 | 2 | Centipeda | 0 | 1 |
| Parabacteroides | 1 | 2 | Chryseolinea | 0 | 1 |
| Gardnerella | 1 | 1 | Cryomorphaceae | 0 | 1 |
| Klugiella | 1 | 1 | Cystobacteraceae | 0 | 1 |
| Odoribacter | 1 | 1 | Extensimonas | 0 | 1 |
| Oscillibacter | 1 | 1 | Frateuria | 0 | 1 |
| Roseburia | 1 | 1 | Halieaceae | 0 | 1 |
| Senegalimassilia | 1 | 1 | Luteibacter | 0 | 1 |
| Thalassospira | 1 | 1 | Massilia | 0 | 1 |
| Acanthopleuribacter | 1 | 0 | Modestobacter | 0 | 1 |
| Aquaspirillum | 1 | 0 | Mumia | 0 | 1 |
| Ardenticatenia | 1 | 0 | Nevskia | 0 | 1 |
| Desulfobacterium | 1 | 0 | Nubsella | 0 | 1 |
| Desulfomonile | 1 | 0 | Pelomonas | 0 | 1 |
| Desulfosarcina | 1 | 0 | Peredibacter | 0 | 1 |
| Elusimicrobia | 1 | 0 | Phenylobacterium | 0 | 1 |
| Erysipelatoclostridium | 1 | 0 | Phyllobacteriaceae | 0 | 1 |
| Erythrobacter | 1 | 0 | Polymorphospora | 0 | 1 |
| Exiguobacterium | 1 | 0 | Portibacter | 0 | 1 |
| Fervidobacterium | 1 | 0 | Rhizobium | 0 | 1 |
| Filomicrobium | 1 | 0 | Rikenella | 0 | 1 |
| Lactococcus | 1 | 0 | Rubrobacter | 0 | 1 |
| Methyloceanibacter | 1 | 0 | Sandaracinaceae | 0 | 1 |
| Oligella | 1 | 0 | Sulfurovum | 0 | 1 |
| Oligotropha | 1 | 0 | Tepidicella | 0 | 1 |
| Parasutterella | 1 | 0 | Thermomarinilinea | 0 | 1 |
| Pseudarcicella | 1 | 0 | Thiogranum | 0 | 1 |
| Roseibacillus | 1 | 0 | Tropicimonas | 0 | 1 |
| Taibaiella | 1 | 0 | Chelatococcus | 216 | 0 |
| Telluria | 1 | 0 | Leuconostoc | 75 | 0 |
| Thiofaba | 1 | 0 | Megasphaera | 39 | 0 |
| Trabulsiella | 1 | 0 | Alteromonas | 36 | 0 |
| Volucribacter | 1 | 0 | Psychrobacter | 33 | 0 |
| Weissella | 1 | 0 | Pedobacter | 32 | 0 |
| Desemzia | 0 | 323 | Proteus | 31 | 0 |
| Rickettsiaceae | 0 | 261 | Filifactor | 24 | 0 |
| Sanguibacter | 0 | 145 | Rheinheimera | 24 | 0 |
| Undibacterium | 0 | 134 | Pleomorphomonas | 21 | 0 |
| Shimazuella | 0 | 120 | Sporosarcina | 17 | 0 |
| Hyphomicrobium | 0 | 74 | Faecalibacterium | 14 | 0 |
| Bosea | 0 | 55 | Sebaldella | 14 | 0 |
| Armatimonadetes | 0 | 50 | Acidibacter | 13 | 0 |
| Prosthecobacter | 0 | 47 | Caulobacter | 13 | 0 |
| Asaccharospora | 0 | 45 | Catenibacterium | 12 | 0 |
| Holophagaceae | 0 | 45 | Erysipelothrix | 12 | 0 |
| Simkaniaceae | 0 | 45 | Sneathia | 11 | 0 |
| Acidimicrobiaceae | 0 | 31 | Fusibacter | 10 | 0 |
| Macromonas | 0 | 31 | Quadrisphaera | 9 | 0 |
| Sulfurihydrogenibium | 0 | 16 | Megamonas | 8 | 0 |
| Planifilum | 0 | 13 | Adhaeribacter | 7 | 0 |
| Stakelama | 0 | 13 | Cardiobacteriaceae | 7 | 0 |
| Millisia | 0 | 9 | Morganella | 7 | 0 |
| Acaricomes | 0 | 7 | Christensenellaceae | 5 | 0 |
| Cruoricaptor | 0 | 5 | Collinsella | 5 | 0 |
| Fodinicola | 0 | 5 | Defluviitaleaceae | 5 | 0 |
| Mucispirillum | 0 | 5 | Ornithinimicrobium | 5 | 0 |
| Devriesea | 0 | 4 | Phascolarctobacterium | 5 | 0 |
| Fontibacillus | 0 | 4 | Cellulomonas | 4 | 0 |
| Anaeroplasma | 0 | 2 | Duganella | 4 | 0 |
| Bdellovibrio | 0 | 2 | Gemmatimonas | 4 | 0 |
| Fibrella | 0 | 2 | Anaerobiospirillum | 3 | 0 |
| Nitrosococcus | 0 | 2 | Bhargavaea | 3 | 0 |
| Pseudonocardia | 0 | 2 | Holdemanella | 3 | 0 |
| Actinobacillus | 0 | 1 | Lentisphaerae | 3 | 0 |
| Allisonella | 0 | 1 | Novosphingobium | 3 | 0 |
| Arcticibacter | 0 | 1 | Promicromonospora | 3 | 0 |
| Azomonas | 0 | 1 | Thermoanaerobacterium | 3 | 0 |
| Azospirillum | 0 | 1 | Acholeplasma | 2 | 0 |
| Caldimonas | 0 | 1 | Desulfomicrobium | 2 | 0 |
| Centipeda | 0 | 1 | Pontibacter | 2 | 0 |
| Chryseolinea | 0 | 1 | Synechococcus | 2 | 0 |
| Cryomorphaceae | 0 | 1 | Venenivibrio | 2 | 0 |
| Cystobacteraceae | 0 | 1 | Acanthopleuribacter | 1 | 0 |
| Extensimonas | 0 | 1 | Aquaspirillum | 1 | 0 |
| Frateuria | 0 | 1 | Ardenticatenia | 1 | 0 |
| Halieaceae | 0 | 1 | Desulfobacterium | 1 | 0 |
| Luteibacter | 0 | 1 | Desulfomonile | 1 | 0 |
| Massilia | 0 | 1 | Desulfosarcina | 1 | 0 |
| Modestobacter | 0 | 1 | Elusimicrobia | 1 | 0 |
| Mumia | 0 | 1 | Erysipelatoclostridium | 1 | 0 |
| Nevskia | 0 | 1 | Erythrobacter | 1 | 0 |
| Nubsella | 0 | 1 | Exiguobacterium | 1 | 0 |
| Pelomonas | 0 | 1 | Fervidobacterium | 1 | 0 |
| Peredibacter | 0 | 1 | Filomicrobium | 1 | 0 |
| Phenylobacterium | 0 | 1 | Lactococcus | 1 | 0 |
| Phyllobacteriaceae | 0 | 1 | Methyloceanibacter | 1 | 0 |
| Polymorphospora | 0 | 1 | Oligella | 1 | 0 |
| Portibacter | 0 | 1 | Oligotropha | 1 | 0 |
| Rhizobium | 0 | 1 | Parasutterella | 1 | 0 |
| Rikenella | 0 | 1 | Pseudarcicella | 1 | 0 |
| Rubrobacter | 0 | 1 | Roseibacillus | 1 | 0 |
| Sandaracinaceae | 0 | 1 | Taibaiella | 1 | 0 |
| Sulfurovum | 0 | 1 | Telluria | 1 | 0 |
| Tepidicella | 0 | 1 | Thiofaba | 1 | 0 |
| Thermomarinilinea | 0 | 1 | Trabulsiella | 1 | 0 |
| Thiogranum | 0 | 1 | Volucribacter | 1 | 0 |
| Tropicimonas | 0 | 1 | Weissella | 1 | 0 |
